# The hidden architecture of back pain: ultrasound-based lumbar multifidus pennation angle analysis - a cross-sectional pilot study

**DOI:** 10.1186/s12891-026-09604-4

**Published:** 2026-03-11

**Authors:** Nagma Sheenam, Nitesh Manohar Gonnade, Ravi Gaur, T K Abins, Arindam Ghosh, Rejuwan Hussain

**Affiliations:** Department of Physical Medicine and Rehabilitation, All India Institute of Medical Sciences, Jodhpur, Rajasthan 342005 India

**Keywords:** Lumbar multifidus, pennation angle, chronic low back pain, muscle architecture, Ultrasound imaging, Muscle thickness, Paraspinal muscles

## Abstract

**Background:**

Chronic low back pain (CLBP) is associated with structural and functional alterations in the lumbar-multifidus (LM) muscle, a key-stabilizer of the spine. In this pilot study, Pennation-angle, a well-established muscle architecture marker not previously assessed in CLBP, was evaluated using ultrasound to compare LM structure between CLBP-patients and healthy-controls and examine its relationship with muscle thickness, pain-intensity and activity-levels.

**Methods:**

This cross-sectional study included 40 adults aged 18–35 years (20 CLBP-patients, 20 healthy-controls) at AIIMS Jodhpur. Bilateral ultrasound-imaging at the L4–L5 level was performed, and the average of right and left measurements was used to quantify superficial and deep LM pennation-angle and muscle-thickness. Pain intensity (NRS-scale) and physical activity levels were recorded. Group comparisons were performed using t-tests or Mann–Whitney U tests; correlations by Pearson’s or Spearman’s coefficients.

**Results:**

CLBP participants showed significantly reduced superficial-pennation angle (7.03 ± 0.98° vs. 8.85 ± 1.25°, *p* < 0.001; Cohen’s *d* = − 1.62), superficial muscle-thickness (1.07 ± 0.187 cm vs. 1.29 ± 0.258 cm, *p* = 0.004; *d* = − 0.96), and deep muscle-thickness (1.18 ± 0.190 cm vs. 1.32 ± 0.190 cm, *p* = 0.026; *d* = − 0.73), while deep-pennation angle showed no group difference (*p* = 0.235; *d* = − 0.38). Superficial-pennation angle correlated positively with superficial muscle-thickness (*r* = 0.678, *p* < 0.001). NRS showed weak negative trends with all LM parameters. Physical activity distributions differed but were statistically non-significant (χ²=2.88, *p* = 0.237), although effect size indicated a small-to-moderate trend toward higher sedentary behaviour in CLBP (Cramer’s V = 0.268).

**Conclusion:**

The observed reduction in LM pennation-angle and muscle-thickness reflects potential architectural compromise and disuse-related atrophy in CLBP. The strong angle–thickness correlation supports the interdependence of muscle size and fiber orientation. Although pain-intensity and activity-levels were not statistically associated to LM morphology, their negative and sedentary trends may still reflect behavioural and pain-related influences on subtle muscle decline. CLBP patients demonstrate distinct LM architectural alterations, emphasizing the value of ultrasound-based assessment and supporting targeted rehabilitation strategies focused on restoring LM function and morphology.

**Institutional ethics committee registration number:**

AIIMS/IEC/2025/5525.

**Supplementary Information:**

The online version contains supplementary material available at 10.1186/s12891-026-09604-4.

## Introduction

**Fig. 1:**
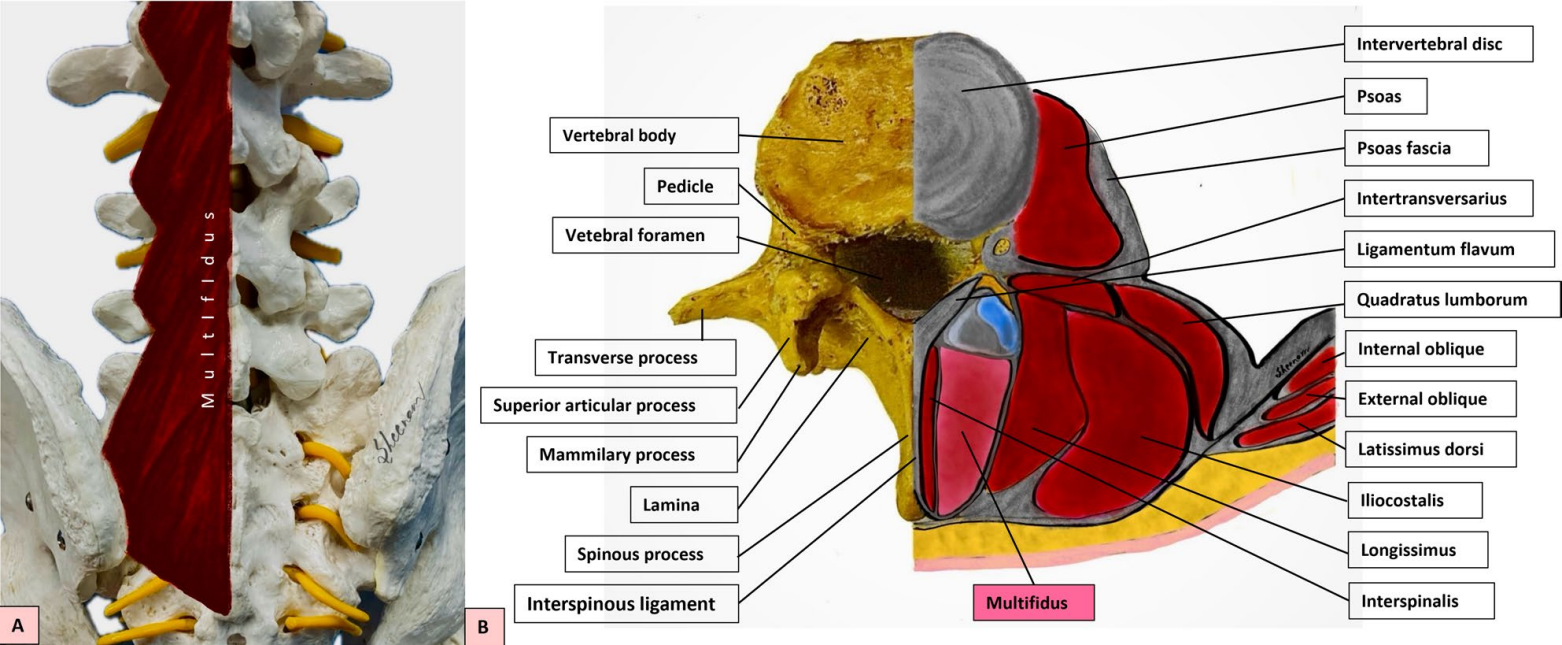
**A**: Posterior view of lumbar multifidus; **B**: Axial lumbar illustration showing multifidus and surrounding anatomical structures. (This figure is an original illustration created by the authors for the present study.)

Low back pain (LBP) is a prevalent and disabling condition affecting a significant portion of the population globally [1, 2]. The lumbar multifidus (LM) muscle, due to its critical role in spinal stability and segmental control, has been extensively studied in the context of LBP [2]. The anatomical orientation and spatial relationships of the lumbar multifidus and adjacent paraspinal structures are illustrated in Fig. 1 (Fig. 1). Structural and functional impairments of the LM, such as atrophy and altered activation patterns, are commonly observed in individuals with chronic low back pain (CLBP) [2–4]. The assessment of LM morphology and function can provide valuable insights into the pathophysiology of LBP and aid in the development of targeted rehabilitation strategies.

Ultrasound imaging (USI) is a reliable, non-invasive tool for evaluating LM muscle architecture, with measurements of cross-sectional area and muscle thickness validated against gold standard methods like magnetic resonance imaging and electromyography [5]. It has been demonstrated to effectively detect LM atrophy and activation patterns in both clinical and research settings [5].

Muscle function is determined by its architectural characteristics, which influence force generation and neuromuscular efficiency. Chronic low back pain is associated with structural and functional alterations of the lumbar multifidus, including reductions in muscle thickness and impaired force-generating capacity. While most ultrasound-based studies in CLBP have focused on muscle size parameters such as thickness or cross-sectional area, these measures do not fully capture architectural features that influence muscle function. Pennation angle, defined as the angle between the muscle fibers and the force-generating axis, is another critical parameter of muscle architecture that influences a muscle’s force-generating capacity. Although the pennation angle has been studied in various muscles, its role in lumbar multifidus architecture, especially in relation to pain severity and layer specific morphological changes in chronic low back pain, remains insufficiently explored.

Recent research has established protocols for measuring the pennation angle of the LM using USI, demonstrating excellent intra-rater reliability [6].

In patients with CLBP, previous studies have shown inconsistent findings regarding LM atrophy and activation. Some studies reported generalized LM atrophy, while others found level and side-specific atrophy [3]. Moreover, altered LM activation patterns during movement have been observed in individuals with induced LBP [7]. Understanding these variations is crucial for developing precise therapeutic interventions. Evidence from other musculoskeletal conditions demonstrates that clinically meaningful improvements in pain and function may coexist with persistent muscle hypotrophy and impaired neuromuscular activation, highlighting the importance of objective muscle assessment beyond patient-reported outcomes [8].

This observational study aims to assess the pennation angle and muscle thickness of the LM in patients with LBP and compare these parameters with healthy controls. The study also seeks to explore the correlation between LM thickness and pennation angle, as well as their association with the Numeric Rating Scale (NRS) score and duration of pain. By establishing normative data and identifying significant differences and correlations, this research can contribute to the optimization of diagnostic and therapeutic approaches for LBP.

This study utilized USI to measure LM pennation angle and thickness, employing established protocols to ensure accuracy and reliability [9, 10].The aim was to compare these morphological parameters between chronic low back pain (CLBP) patients and healthy controls, and to explore their relationship with pain intensity in CLBP. The findings are intended to enhance understanding of lumbar multifidus architecture and support improved clinical assessment and targeted intervention strategies.

## Methods

### Study design and participants

This cross-sectional pilot observational study was conducted in the Department of Physical Medicine and Rehabilitation at AIIMS Jodhpur to compare lumbar multifidus (LM) muscle morphology between individuals with chronic low back pain (CLBP) and healthy controls. As an exploratory pilot study, the sample size was selected pragmatically to assess feasibility and to generate preliminary estimates of variability and effect size for planning future adequately powered studies. A total of 40 adults aged 18–35 years were recruited, comprising 20 participants with non-specific unilateral CLBP of more than three months’ duration and pain intensity of at least 4/10 on the Numeric Rating Scale, along with 20 healthy controls with no history of low back pain. Individuals were excluded if they had pregnancy or breastfeeding, prior lumbar surgery, spinal trauma or fracture, structural spinal abnormalities, systemic inflammatory disease, severe neurological deficits, recent corticosteroid use, competitive athletic training, or any skin disease over the imaging site. All participants provided written informed consent, and demographic variables including age, sex, height, weight, body mass index (BMI), occupation, and pain duration (for CLBP) were recorded. Physical activity was categorized using self-reported daily and occupational activity patterns and classified as Sedentary, Light–Moderate, or Vigorous according to the World Health Organization (WHO) 2020 Guidelines on Physical Activity and Sedentary Behaviour.

### Ultrasound assessment of Lumbar Multifidus (LM) morphology

**Fig. 2:**
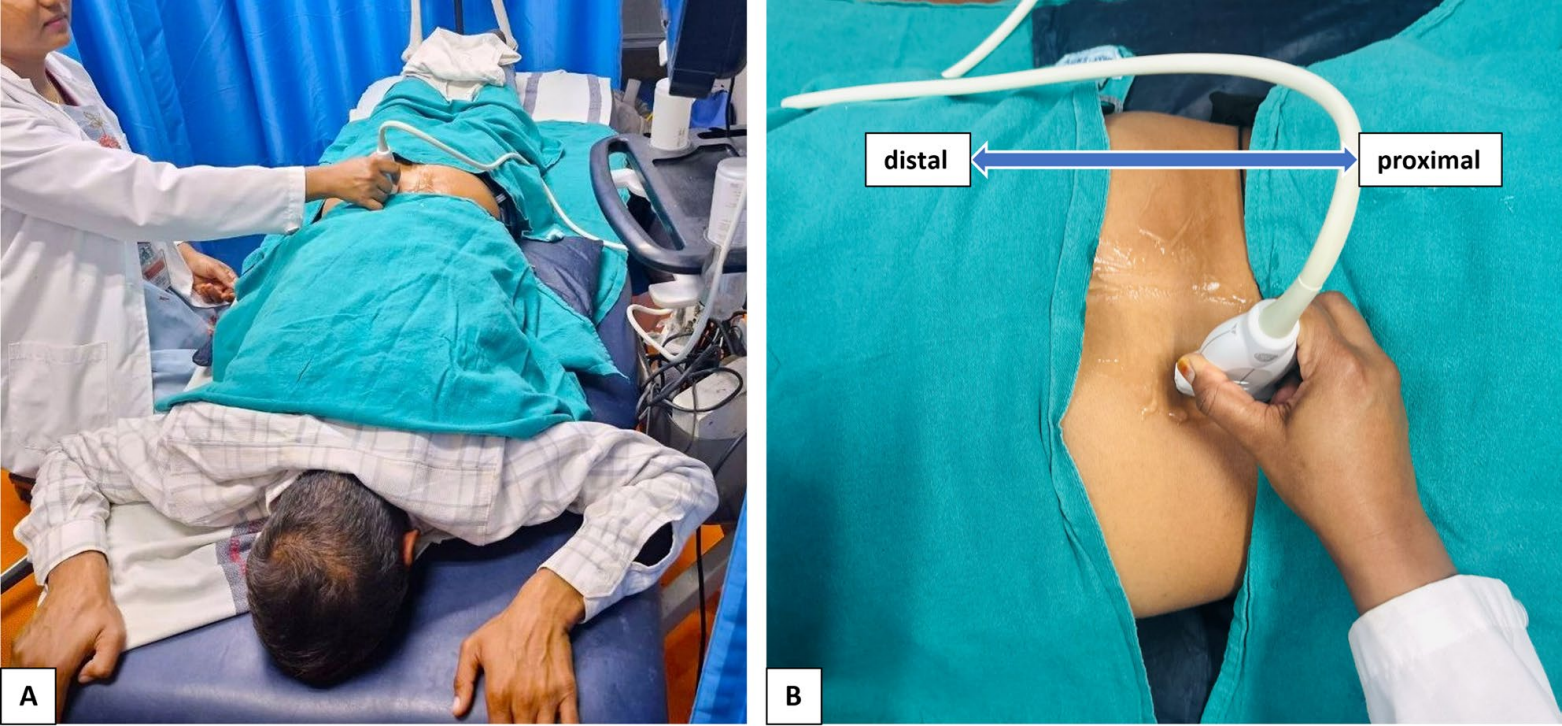
**A**: Patient positioned prone for lumbar ultrasound imaging, with a pillow placed under the abdomen to reduce lumbar lordosis to < 10°, and the arms abducted to 90° with elbows flexed; **B**: Probe placement technique during right multifidus ultrasound image acquisition

**Fig. 3:**
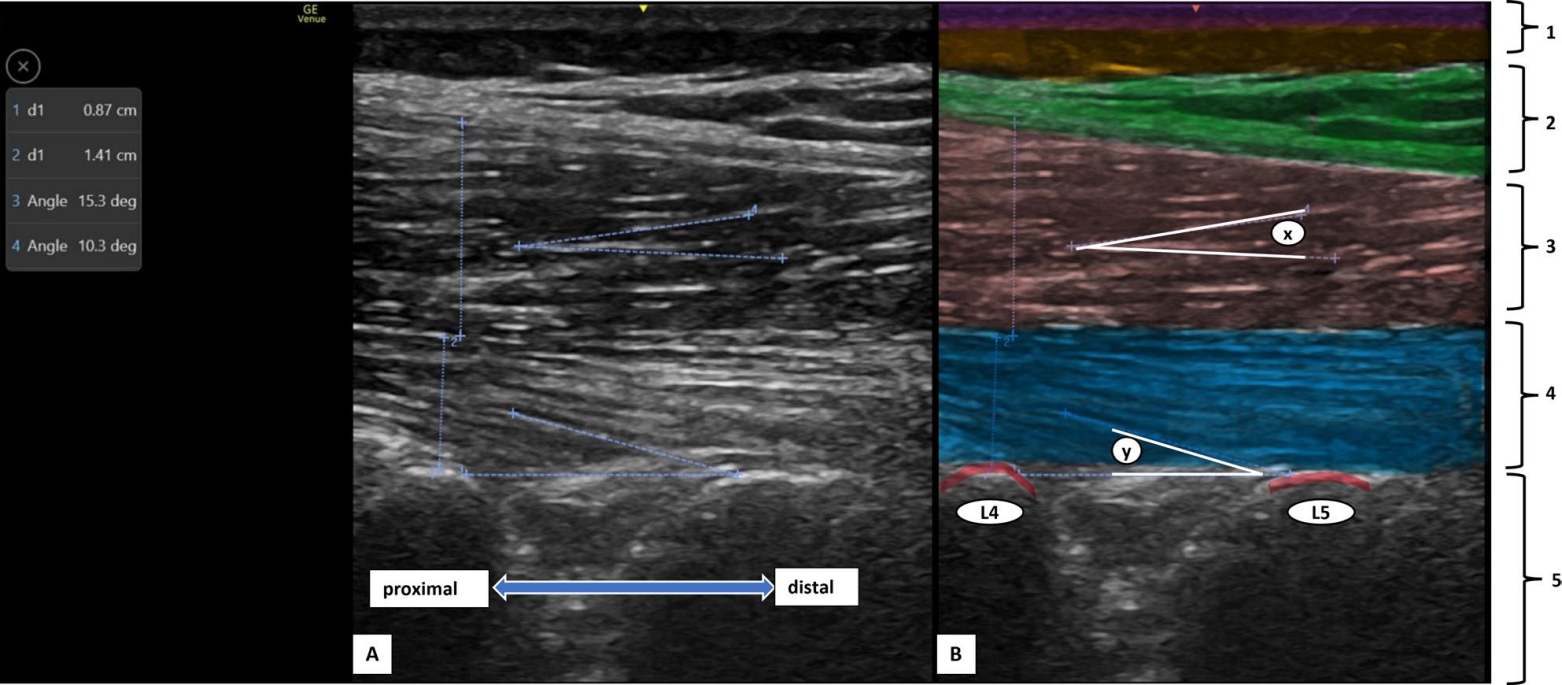
**A**: Ultrasound sagittal image of the lumbar multifidus demonstrating measurements of muscle thickness (d1, d2) and pennation angles for the superficial and deep fibers (X and Y, respectively); **B**: Color-enhanced ultrasound image illustrating the anatomical layers overlying the multifidus, including: (1) skin, subcutaneous fat (2), thoracolumbar fascia (3), superficial multifidus (4), deep multifidus (5), laminae of L4–L5 vertebrae. Labels X and Y represent the superficial and deep pennation angle measurement vectors, respectively

Ultrasound imaging was performed by a single trained examiner who was blinded to participant group allocation (CLBP or healthy control), using a Venue Go R3 device, with standardized depth and gain settings kept constant for all participants throughout image acquisition. To minimise variability related to probe pressure, the transducer was applied with light, consistent contact using adequate coupling gel and careful probe orientation, avoiding visible tissue compression, in accordance with published practical guidance for lumbar multifidus ultrasound imaging [5]. Probe positioning and measurement procedures followed a predefined protocol to ensure consistency and reproducibility across examinations. Participants were positioned prone with the lumbar spine flattened to less than 10° of lordosis using a pillow under the abdomen, with the arms abducted to 90° and elbows flexed (Fig. 2). The ultrasound probe was positioned by first locating the lumbar spinous process, moving laterally to identify the vertebral lamina, then scanning caudally toward the sacrum and cranially to the L4–L5 level to ensure consistent image acquisition. A single high-quality image was obtained on each side, and the average of bilateral measurements was used for analysis. Pennation angle was defined as the angle between the most hyperechoic multifidus fiber and a baseline joining the laminae at L4–L5. Muscle thickness was measured from the L4 lamina to the superior border of the deep LM for deep thickness, and from the thoracolumbar fascia to the inferior border of the superficial LM for superficial thickness [6] (Fig. 3).

All analyses were performed using SPSS version 26. Each assessments was completed in a single session by the same trained examiner to ensure consistency. The primary outcomes were pennation angle and muscle thickness values of the superficial and deep LM. Secondary measures included pain intensity by 11-point Numeric Rating Scale (NRS) [11], and physical activity level. Statistical analyses were conducted after assessing normality with the Shapiro–Wilk test. Independent Samples t-tests or Mann–Whitney U tests were used to compare morphological variables between groups. For all primary between-group comparisons, mean values, effect sizes, and corresponding 95% confidence intervals (CIs) were calculated and reported to convey the precision and compatibility of the estimated effects, in accordance with recommended statistical reporting practices [12]. Correlation analysis was performed to assess relationships between lumbar multifidus architectural parameters and pain intensity. Pearson’s correlation was used for normally distributed variables, specifically to examine relationships between pennation angle and muscle thickness, whereas Spearman’s correlation assessed associations between LM morphology and pain intensity within the CLBP group when normality assumptions were not met. Differences in physical activity levels between groups were analysed using the chi-square test, and statistical significance was set at *p* < 0.05. Physical activity was assessed as a categorical variable to describe habitual activity patterns and was not included in correlation analyses with lumbar multifidus morphological parameters. Given the exploratory pilot nature of the study, no formal correction for multiple comparisons was applied; therefore, results are interpreted descriptively with emphasis on effect sizes and overall patterns rather than confirmatory inference. Effect sizes were reported alongside *p*-values to aid clinical interpretation. For group differences, Cohen’s *d* was interpreted using physiotherapy-specific thresholds, where small, medium, and large effects correspond to approximately 0.1, 0.4, and 0.8, respectively. For associations, Pearson’s *r* and Spearman’s ρ were interpreted using thresholds of approximately 0.3 (small), 0.5 (medium), and 0.6 (large), as recommended by Zieliński et al. [13].

## Results

A total of 40 participants were included, with 20 in the CLBP group and 20 in the healthy group. The median age was significantly higher in the CLBP group *[33.0 years (27.0–35.0)]* compared to the healthy group *[27.5 years (23.8–29.0)]* (*p* = 0.012). Height, weight and BMI did not differ significantly between the groups (Table 1).

**Table 1:** Baseline sociodemographic characteristics of participants

Variable	CLBP (*N* = 20)	Healthy (*N* = 20)	*p*-value
Age (years)*	33.0 (27.0–35.0)	27.5 (23.8–29.0)	0.012
Height (cm)*	165 (161–168)	166 (157–174)	0.607
Weight (kg) **	65.0 ± 13.7	68.1 ± 15.0	0.506
BMI (kg/m²)**	24.1 ± 4.77	24.8 ± 5.18	0.638

*median (Q1–Q3), Mann–Whitney U test; ** mean ± SD, Independent *t*-test

The distribution of physical activity levels differed between the CLBP and healthy groups. Sedentary behaviour was more common in individuals with CLBP (25%) compared to healthy participants (10%). Conversely, light-to-moderate activity was more frequent in the healthy group (80%) compared to the CLBP group (55%). Vigorous activity showed a similar distribution in both groups (20% vs. 10%). A chi-square test of independence indicated that the association between physical activity level and group was *not statistically significant* (χ² (2) = 2.88, *p* = 0.237). However, the effect size (Cramer’s V = 0.268) suggested a *small-to-moderate association*, reflecting differences in the distribution of activity levels between groups, which are therefore presented descriptively. (Table 2).

**Table 2:** Comparison between physical activity levels between CLBP and healthy groups

Physical Activity Level	CLBP^a^ (*n* = 20)	Healthy^a^ (*n* = 20)	χ²	*p*-value	Cramer’s V
Sedentary	25% (5)	10% (2)	2.88	0.237	0.268
Light–Moderate	55% (11)	80% (16)			
Vigorous	20% (4)	10% (2)			

a: (% frequency)n; N: Total number of participants = 40; *χ²: Chi-Square Analysis*

Regarding lumbar multifidus morphology, individuals with CLBP demonstrated significantly reduced superficial pennation angle compared with healthy controls (7.03 ± 0.98° vs. 8.85 ± 1.25°, 95% CI 6.57–7.49 vs. 8.27–9.44; *p* < 0.001), with a large effect size (Cohen’s *d* = − 1.62), indicating a clinically meaningful architectural difference. Superficial muscle thickness was also significantly lower in the CLBP group (1.07 ± 0.187 cm vs. 1.29 ± 0.258 cm, 95% CI 0.98–1.16 vs. 1.17–1.41; *p* = 0.004), corresponding to a large effect size (*d* = − 0.96).

Deep muscle parameters showed similar trends: deep muscle thickness was significantly reduced in the CLBP group (1.18 ± 0.190 cm vs. 1.32 ± 0.190 cm, 95% CI 1.09–1.27 vs. 1.23–1.41; *p* = 0.026), with a moderate effect size (*d* = − 0.73). However, deep pennation angle did not differ significantly between groups (*p* = 0.235). Overall, individuals with chronic low back pain exhibited reduced lumbar multifidus muscle thickness and smaller superficial pennation angles, indicating morphological alterations compared to healthy individuals (Table 3).

**Table 3:** Comparison of lumbar multifidus muscle morphology between CLBP and healthy groups

Variable	CLBP (*N* = 20)		Healthy (*N* = 20)		*p*-value **	effect size^d^
	Mean ± SD	95% CI*	Mean ± SD	95% CI*		
Superficial pennation angle (°)	7.03 **±** 0.982	6.57–7.49	8.85 **±** 1.25	8.27–9.44	< 0.001	-1.62
Deep pennation angle (°)	8.38 **±** 2.27	7.32–9.44	9.23 **±** 2.17	8.21–10.20	0.235	-0.38
Superficial muscle thickness (cm)	1.07 **±** 0.187	0.98–1.16	1.29 **±** 0.258	1.17–1.41	0.004	-0.96
Deep muscle thickness (cm)	1.18 **±** 0.190	1.09–1.27	1.32 **±** 0.190	1.23–1.41	0.026	-0.73

*(95% confidence intervals calculated assuming a *t*-distribution with *N* − 1 degrees of freedom); **Independent Samples T-Test; d: Cohen’s d; *N* = 20 patients in each group

Correlation analysis showed significant associations between muscle pennation angles and muscle thickness measurements of the lumbar multifidus. A moderate positive correlation was observed between the superficial pennation angle and superficial muscle thickness (*r* = 0.678, *p* < 0.001), indicating that greater muscle thickness was associated with larger pennation angles. The deep pennation angle was weakly but significantly correlated with deep muscle thickness (*r* = 0.330, *p* = 0.037), suggesting a mild association between deep architectural alignment and muscle mass. Overall, these findings indicate that muscle thickness and pennation angle are positively related, with thicker lumbar multifidus fibers demonstrating larger pennation angles, particularly in the superficial region (Table 4).

**Table 4:** Pearson correlation between pennation angles and muscle thickness variables (*N* = 40)

	Variables		Pearson Correlation	*p*-value
1	Superficial pennation angle (°)	Superficial muscle thickness (cm)	0.678	< 0.001
2	Deep pennation angle (°)	Deep muscle thickness (cm)	0.330	0.037

N: Total number of participants = 40

Pain intensity in the CLBP group showed a median NRS score of 7.0 (5.75, 8.0). The Shapiro–Wilk test indicated that NRS values were non-normally distributed (W = 0.882, *p* = 0.019), and therefore Spearman correlations were used.

Within the CLBP group, NRS demonstrated weak negative correlations with all four lumbar multifidus morphology parameters. None of these associations reached statistical significance (all *p* > 0.05), and therefore no definitive relationship between pain intensity and lumbar multifidus architecture can be inferred from these data. The results are presented descriptively without implication of clinical significance (Table 5).

**Table 5:** Spearman correlation between NRS pain intensity and multifidus morphology (CLBP Group),** (** *N* = 20 CLBP patients)

Morphology Variable	Spearman’s ρ	*p*-value
Superficial pennation angle (°)	–0.424	0.062
Deep pennation angle (°)	–0.252	0.284
Superficial muscle thickness (cm)	–0.135	0.569
Deep muscle thickness (cm)	–0.426	0.061

## Discussion

The present study compared LM muscle morphology, specifically PA and muscle thickness, between individuals with CLBP and healthy controls, using USI. The findings provide important insights into LM architectural alterations associated with persistent low back pain and support the growing evidence that LM dysfunction plays a central role in spinal stability and pain pathology [2–4].

Consistent with our primary hypothesis, lumbar multifidus architecture differed between individuals with chronic low back pain and healthy controls, demonstrating large effect sizes for superficial pennation angle and superficial muscle thickness and a moderate effect size for deep muscle thickness, shows clinically meaningful architectural differences. These findings align with earlier literature establishing that CLBP is frequently accompanied by LM atrophy, architectural disruption, and impaired activation patterns [3, 4]. Danneels et al. demonstrated level- and side-specific LM atrophy in CLBP patients, suggesting that chronic pain and disuse provoke localized structural degeneration [3]. Notably, subsequent ultrasonographic evidence indicates that these morphological and functional impairments are most consistently detected at the lower lumbar segments, particularly around the L5 level, where significant reductions in multifidus cross-sectional area and voluntary contraction have been observed, while more cranial levels often remain relatively preserved [14]. The decreased LM muscle thickness observed in our findings is consistent with previous reports of lumbar multifidus morphological alterations in individuals with chronic low back pain and suggests an association with chronic pain status and reduced functional loading [2].

A key contribution of this study is the assessment of LM pennation angle, a relatively novel yet functionally meaningful muscle architecture parameter. Previous work has highlighted PA as an important determinant of muscle force production due to its influence on fiber orientation and fascicle arrangement [15]. Monsalve-Vicente et al. demonstrated the reliability of LM PA measurement via ultrasound, providing a methodological foundation for subsequent exploratory and clinical research [6]. The current study extends this knowledge by demonstrating that superficial PA is significantly reduced in CLBP, suggesting that chronic pain and reduced activation are associated with changes in both muscle size and fiber orientation.

The strong positive correlation observed between LM pennation angle and muscle thickness further supports the architectural interdependence that has been reported in earlier research. Monsalve-Vicente et al. showed that larger muscle thickness is typically associated with increased pennation, reflecting an adaptive relationship between fascicle angle and cross-sectional area [6]. Our findings reflect similar behaviour, suggesting that reductions in LM bulk may be accompanied by diminished fiber angulation, thereby compromising the muscle’s ability to generate optimal force. This relationship is also consistent with multiscale muscle adaptation theory, which posits that hypertrophy increases pennation through geometric remodeling, while underloading and atrophy reduce fiber diameter and thus decrease pennation [16].

Interestingly, deep PA did not differ significantly between groups, which may indicate that superficial LM fibers, which are more involved in dynamic trunk stabilization, may be more susceptible to disuse and pain- related changes. Histological evidence from Sirca and Kostevc demonstrated a higher proportion and larger diameter of Type I fibers in the deep lumbar multifidus compared with the superficial layer, supporting greater fatigue resistance of the deeper fibers [17]. In addition, electromyographic findings by Moseley et al. showed that superficial fibers exhibit direction dependent, phasic activation related to spinal orientation, whereas deep fibers demonstrate more tonic, task independent activity [18]. Together, these findings suggest that the more movement dependent superficial fibers may be more vulnerable to reduced loading or disuse, resulting in preferential architectural changes such as reduced pennation angle, while the tonically active deep layer may be relatively preserved despite chronic symptoms. From a rehabilitation perspective, this layer-specific adaptation supports the clinical emphasis on exercise strategies that selectively enhance activation, coordination, and loading of the superficial lumbar multifidus, including motor control training, task-specific stabilization exercises, and progressive functional loading, while maintaining the stabilizing role of the deep fibers. This pattern aligns with prior evidence that LM impairment in CLBP may be selective, with superficial regions exhibiting more pronounced functional and structural changes [9]. Although participants were recruited within a restricted young adult age range, the statistically higher age observed in the CLBP group may represent a source of subtle residual confounding and should be cautiously considered when interpreting differences in lumbar multifidus architecture.

Our findings align with recent evidence indicating that chronic low back pain is associated with structural adaptations of paraspinal tissues. Ultrasound imaging has demonstrated that individuals with CLBP may exhibit paraspinal muscle hypotrophy with a concave muscle profile, accompanied by thoracolumbar fascia thickening, particularly with longer symptom duration, highlighting the potential influence of chronicity, posture, and age related factors on muscle morphology [19]. At the same time, structural abnormalities detected on ultrasound do not necessarily imply functional impairment. Case based evidence shows that ultrasound can identify subtle lumbar structural lesions, such as isolated transverse process fractures missed on conventional radiography, which may remain clinically stable and underscore the need to interpret imaging findings in conjunction with clinical context [20].

Pain intensity was not significantly associated with lumbar multifidus morphology in this study. Although weak negative correlations were observed, these findings should be interpreted with caution, as they may reflect random variation related to limited statistical power or the absence of a true linear relationship. While non-significant results, this pattern supports previous work indicating that chronic nociception may lead to neuromuscular inhibition, reduced activation, and gradual structural compromise [2, 7]. Importantly, contemporary pain adaptation models indicate that pain severity in chronic low back pain does not necessarily correspond to peripheral muscle structure, as pain perception is influenced by central sensitization, motor adaptation, and psychosocial factors [21]. In knee osteoarthritis, for example, radiographic severity has been shown to correlate poorly with systemic inflammatory burden, reinforcing that symptom experience and structural adaptation may evolve through partially independent mechanisms [22]. Accordingly, morphological alterations of the lumbar multifidus may represent protective or compensatory responses that can persist independently of pain intensity, reflecting the multifactorial and centrally modulated nature of chronic pain [23, 24].

The absence of strong statistical associations may be due to the relatively small sample size inherent to this pilot design, the multifactorial nature of CLBP, and the possibility that structural changes may not correlate linearly with pain experiences.

Physical activity patterns revealed a non-significant trend toward higher sedentary behaviour in CLBP participants. Although not statistically significant, the effect size suggests meaningful behavioural differences between groups that may contribute to muscle disuse and architectural alterations. Reduced activity levels and fear-avoidant behaviours are well-recognized contributors to LM atrophy and dysfunctional motor control in CLBP populations [7].

Ultrasound proved to be a practical and reliable modality for assessing both LM thickness and pennation angle in this study, in agreement with earlier research supporting its accuracy and reproducibility [5, 6, 10]. Nevertheless, ultrasound-based measurements remain sensitive to technical factors such as probe orientation, operator experience, and imaging conditions. In addition, all measurements were obtained in a standardized prone position, which may influence muscle architecture compared with functional or weight-bearing postures. These factors should be considered when interpreting the observed architectural differences and highlight the importance of standardized protocols and cautious comparison across studies. The ability to capture subtle changes in muscle morphology at the bedside reinforces its value as a routine clinical assessment tool for monitoring LM health, guiding rehabilitation progress, and individualizing intervention plans.

Overall, the findings of this study support the hypothesis that CLBP is associated with significant LM architectural disruption, particularly in the superficial fibers, and contribute novel evidence using PA as a marker of muscle function. The integration of PA with conventional thickness measurements may enhance the diagnostic value of USI and provide clinicians with deeper insight into the structural underpinnings of chronic spinal pain. These findings highlight the need for targeted rehabilitation strategies, such as motor control training, multifidus activation therapy, and progressive loading programs, to restore LM morphology and improve spinal stability in CLBP patients. In addition, future rehabilitation approaches may benefit from incorporating intelligent, data driven technologies that integrate lower limb biomechanics, gait characteristics, and trunk muscle function to address kinetic chain interactions and enable more personalized rehabilitation strategies [25, 26].

Although magnetic resonance imaging allows precise quantification of paraspinal muscle morphology, its routine use for serial monitoring of lumbar multifidus architecture is limited by high cost, limited accessibility, and poor feasibility for repeated follow up assessments, particularly in resource constrained healthcare settings. Ultrasound imaging offers a pragmatic alternative, enabling real time bedside evaluation of muscle thickness and pennation angle with good reliability and minimal patient burden. This advantage is especially relevant in chronic low back pain, where longitudinal monitoring of muscle architecture during rehabilitation is clinically desirable. By incorporating pennation angle, a parameter reflecting fiber orientation and force generation capacity, alongside conventional thickness measures, ultrasound based assessment provides a more functionally meaningful evaluation of lumbar multifidus health that is both scalable and applicable in routine clinical practice.

## Conclusion

CLBP patients exhibit clear architectural alterations of the lumbar multifidus, characterized by reduced superficial pennation angle and superficial and deep muscle thickness, with medium to large effect sizes indicating an association between chronic low back pain and altered muscle morphology. The significant correlation between pennation angle and muscle thickness highlights the close relationship between fiber orientation and muscle bulk. While pain intensity and activity levels did not show statistical associations, their consistent negative and sedentary trends suggest underlying behavioural and nociceptive influences on muscle architecture. Overall, these findings underscore the clinical value of ultrasound-based assessment of the multifidus and support the need for targeted rehabilitation strategies aimed at restoring muscle morphology and enhancing spinal stability in individuals with chronic low back pain. As these observations arise from a pilot cohort of young adults, their broader applicability should be confirmed in larger and older populations.

### Limitations

While the study offers valuable insights through a comprehensive clinical evaluation, its cross-sectional design limits causal interpretation. The pilot sample size may have limited statistical power to detect modest associations and restricts broader generalisability. The narrow age range of participants (18–35 years), chosen to minimise age related effects on muscle morphology, may limit the generalisability of the findings to older populations with chronic low back pain. Additionally, Despite recruitment within a narrow age range, residual age related confounding and differences in physical activity which were descriptively analysed but not adjusted for due to the pilot sample size cannot be excluded, and the findings should be interpreted as associative rather than adjusted effects. Although formal intra-rater reliability analysis was not performed within the present sample, measurements were obtained by an experienced examiner using a standardized protocol supported by previously published reliability data; however, future investigations will further strengthen methodological rigor by incorporating study-specific reliability assessments. The use of ultrasound provided practical assessment of muscle morphology, though advanced imaging or biomarkers could have offered deeper structural and neurophysiological insights. In addition, the absence of direct muscle function or activation assessment, such as electromyography or functional performance measures, limits the ability to relate architectural changes to functional impairment. Bilateral lumbar multifidus measurements were averaged to allow group level comparison with healthy controls, which may have obscured side to side differences in unilateral CLBP. Future studies should include longitudinal and interventional designs to evaluate changes in lumbar multifidus architecture over time and in response to targeted rehabilitation, and include functional and neuromuscular assessments to better relate structural findings to clinical outcomes.

## Supplementary Information

Supplementary Material 1.

## Data Availability

Data is provided as supplementary information files.

## Abbreviations

CLBP: Chronic Low Back Pain
LM: Lumbar Multifidus
USI: Ultrasound Imaging
PA: Pennation Angle
NRS: Numeric Rating Scale
BMI: Body Mass Index

## References

[1] Wu A, March L, Zheng X, Huang J, Wang X, Zhao J, et al. Global low back pain prevalence and years lived with disability from 1990 to 2017: estimates from the global burden of disease study 2017. Ann Transl Med. 2020;8(6):299. 32355743 10.21037/atm.2020.02.175 PMC7186678

[2] Naghdi N, Mohseni-Bandpei MA, Taghipour M, Rahmani N. Lumbar multifidus muscle morphology changes in patient with different degrees of lumbar disc herniation: an ultrasonographic study. Med (Mex). 2021;57(7):699. 10.3390/medicina57070699 PMC8307190 34356981

[3] Danneels LA, Vanderstraeten GG, Cambier DC, Witvrouw EE, De Cuyper HJ. CT imaging of trunk muscles in chronic low back pain patients and healthy control subjects. Eur Spine J Off Publ Eur Spine Soc Eur Spinal Deform Soc Eur Sect Cerv Spine Res Soc. 2000;9(4):266–72. 10.1007/s005860000190 PMC3611341 11261613

[4] Kjaer P, Bendix T, Sorensen JS, Korsholm L, Leboeuf-Yde C. Are MRI-defined fat infiltrations in the multifidus muscles associated with low back pain? BMC Med. 2007;5:2. 17254322 10.1186/1741-7015-5-2 PMC1796893

[5] Stokes M, Rankin G, Newham DJ. Ultrasound imaging of lumbar multifidus muscle: normal reference ranges for measurements and practical guidance on the technique. Man Ther. 2005;10(2):116–26. 15922232 10.1016/j.math.2004.08.013

[6] Monsalve-Vicente C, Muñoz-Zamarro D, Cuenca-Zaldívar N, Fernández-Carnero S, Selva-Sarzo F, Nunez-Nagy S, et al. Architectural ultrasound pennation angle measurement of lumbar multifidus muscles: A reliability study. J Clin Med. 2022;11(17):5174. 36079105 10.3390/jcm11175174 PMC9457246

[7] Shih HJS, Van Dillen L, Kutch J, Kulig K. Individuals with recurrent low back pain exhibit further altered frontal plane trunk control in remission than when in pain. Clin Biomech Bristol Avon. 2021;87:105391. 10.1016/j.clinbiomech.2021.105391 PMC8392132 34118490

[8] Sheenam N, Gaur R, Gonnade NM, Dixit A, TK A. Knee functional outcomes and quadriceps hypotrophy after ACL reconstruction: a prospective observational study. BMC Sports Sci Med Rehabilitation. 2025;17(1):120. 10.1186/s13102-025-01055-z PMC12065330 40349070

[9] Duffy N, O’Sullivan C, Kelly G. Multifidus muscle size and percentage thickness changes among patients with unilateral chronic low back pain (CLBP) and healthy controls in prone and standing. Man Ther. 2014;19(5):433–9. 10.1016/j.math.2014.04.009 24909431

[10] Sánchez Romero EA, Alonso Pérez JL, Muñoz Fernández AC, Battaglino A, Castaldo M, Cleland JA, et al. Reliability of sonography measures of the lumbar multifidus and transversus abdominis during static and dynamic activities in subjects with Non-Specific chronic low back pain. Diagnostics. 2021;11(4):632. 33915766 10.3390/diagnostics11040632 PMC8065451

[11] Hawker GA, Mian S, Kendzerska T, French M. Measures of adult pain: visual analog scale for pain (vas pain), numeric rating scale for pain (nrs pain), Mcgill pain questionnaire (mpq), short-form Mcgill pain questionnaire (sf‐mpq), chronic pain grade scale (cpgs), short form‐36 bodily pain scale (sf‐36 bps), and measure of intermittent and constant osteoarthritis pain (icoap). Arthritis Care Res. 2011;63(S11):S240–52. 10.1002/acr.20543 22588748

[12] Andrade C. How to understand the 95% confidence interval around the relative risk, odds ratio, and hazard ratio. J Clin Psychiatry. 2023;84(3):23f14933. 10.4088/JCP.23f14933 37256636

[13] Zieliński G. Effect size guidelines for individual and group differences in physiotherapy. Arch Phys Med Rehabil. 2025;106(12):1844–9. 10.1016/j.apmr.2025.05.013 40543698

[14] Wallwork TL, Stanton WR, Freke M, Hides JA. The effect of chronic low back pain on size and contraction of the lumbar multifidus muscle. Man Therap. 2009;14(5):496–500. 19027343 10.1016/j.math.2008.09.006

[15] Trevino M, Perez S, Sontag S, Olmos A, Jeon S, Richardson L. Influence of pennation angle and muscle thickness on mechanomyographic Amplitude–Torque relationships and Sex-Related differences in the Vastus lateralis. J Funct Morphol Kinesiol. 2023;8(2):53. 37218849 10.3390/jfmk8020053 PMC10204520

[16] Wisdom KM, Delp SL, Kuhl E, Review. Use it or lose it: multiscale skeletal muscle adaptation to mechanical stimuli. Biomech Model Mechanobiol. 2015;14(2):195–215. 25199941 10.1007/s10237-014-0607-3 PMC4352121

[17] Sirca A, Kostevc V. The fibre type composition of thoracic and lumbar paravertebral muscles in man. J Anat. 1985;141:131. 2934358 PMC1166395

[18] Moseley GL, Hodges PW, Gandevia SC. Deep and superficial fibers of the lumbar multifidus muscle are differentially active during voluntary arm movements. Spine. 2002;27(2):E29–36. 11805677 10.1097/00007632-200201150-00013

[19] Vita F, Donati D, Pederiva D, Stella SM, Tedeschi R, Miceli M, Faldini C, Galletti S. Classification of paravertebral muscle trophism and its correlation with thoraco-lumbar fascia thickening in patients with chronic low back pain. J Ultrasound. 2025;28(3):621–6 . 10.1007/s40477-025-00988-y PMC12496364 39841352

[20] Biglia A, Morandi V, Maggiore F, Castello LM, Donati D, Tedeschi R, Langone L, Vita F, Bonacchi G, Stella SM, Ciampi B. Ultrasound detection of isolated lumbar transverse process fractures: a case series. J Ultrasound. 2025;1–8. 10.1007/s40477-025-01009-8 PMC13000063 40410640

[21] Sheenam N, Gaur R, Gonnade NM, Abins TK, Ghosh A, Hussain R. Central sensitisation in chronic low back pain: A cross-sectional study. Indian J Anaesth. 2025;69(10):1033–8. 40979767 10.4103/ija.ija_433_25 PMC12445777

[22] Abins TK, Veluguleti U, Sheenam N, Kumar S, Gaur N, Gosh A, Hussain R, Gonnade N, Gaur R, Sarankumar G. Gonnade II N. Association between systemic inflammation and radiographic severity in knee osteoarthritis: A trial baseline cohort analysis. Cureus. 2025;17(9):e93371. 10.7759/cureus.93371 PMC12559018 41164045

[23] Hodges PW, Tucker K. Moving differently in pain: a new theory to explain the adaptation to pain. Pain. 2011;152(3):S90–8. 21087823 10.1016/j.pain.2010.10.020

[24] Hodges PW. Pain and motor control: from the laboratory to rehabilitation. J Electromyogr Kinesiol. 2011;21(2):220–8. 21306915 10.1016/j.jelekin.2011.01.002

[25] Lu Z, Li X, Sun D, Song Y, Fekete G, Kovács A, Gao Z, Zheng J, Xiang L, Gu Y. Computationally tuned dual-layer lattice pads adapted to gait-induced pressure distribution. Npj Adv Manuf. 2025;2(1):43.

[26] Xu X, Sekiguchi Y, Honda K, Izumi SI. Motion analysis of 3D multi-segmental spine during gait in symptom remission people with low back pain: a pilot study. BMC Musculoskelet Disord. 2025;26(1):269. 40097987 10.1186/s12891-025-08506-1 PMC11912730

