# Supplementary material for: The hidden architecture of back pain: ultrasound-based lumbar multifidus pennation angle analysis - a cross-sectional pilot study
Source: BMC Musculoskelet Disord. 2026 Mar 11;27:337. doi: 10.1186/s12891-026-09604-4 (PMC13088599; doi:10.1186/s12891-026-09604-4)
Supplement: Supplementary file 1 — Supplementary Material 1. [file 12891_2026_9604_MOESM1_ESM.docx]

**SUPPLEMENTARY TABLE** [1](file:///E:\Research\chinchu's\chinchu%20paper%20BMC.docx#_bookmark11)

| S.  No. | Group | Age | Gender | **Body Mass Index**  **(kg/m²)** | Level_of_activity | NRS | Average superficial LM Pennation angle **(°)** | Average deep LM Pennation angle **(°)** | Average superficial LM muscle thickness **(cm)** | Average deep LM muscle thickness **(cm)** |
| --- | --- | --- | --- | --- | --- | --- | --- | --- | --- | --- |
| 1 | CLBP | 33 | M | 30.84 | low-moderate | 7 | 5.72 | 6.3 | 0.98 | 0.98 |
| 2 | CLBP | 27 | F | 28.31 | low-moderate | 6 | 5.45 | 6.9 | 0.81 | 1.155 |
| 3 | CLBP | 35 | M | 23.31 | heavy | 8 | 7.85 | 7 | 1.255 | 0.925 |
| 4 | CLBP | 18 | M | 18.83 | Sedentary | 8 | 6.35 | 6.4 | 1.39 | 1.07 |
| 5 | CLBP | 25 | M | 24.61 | Sedentary | 8 | 6.95 | 8.4 | 1.015 | 1.12 |
| 6 | CLBP | 35 | M | 30.37 | Sedentary | 3 | 7.47 | 8.35 | 1.06 | 1.48 |
| 7 | CLBP | 35 | M | 23.31 | low-moderate | 7 | 6.25 | 8.35 | 0.785 | 1.285 |
| 8 | CLBP | 33 | M | 26.99 | Sedentary | 5 | 7.635 | 13.3 | 1.205 | 1.095 |
| 9 | CLBP | 35 | M | 20.31 | low-moderate | 5 | 8.475 | 6.9 | 1.17 | 1.425 |
| 10 | CLBP | 27 | M | 27.89 | Sedentary | 6 | 7.65 | 10.25 | 1.195 | 1.36 |
| 11 | CLBP | 18 | F | 14.02 | low-moderate | 8 | 6.1 | 8.2 | 1.085 | 0.8 |
| 12 | CLBP | 33 | F | 23.15 | low-moderate | 8 | 5.95 | 8.2 | 0.705 | 1.315 |
| 13 | CLBP | 34 | F | 21.62 | low-moderate | 6 | 7.565 | 12.4 | 1.045 | 1.225 |
| 14 | CLBP | 27 | F | 23.88 | low-moderate | 8 | 6.15 | 4.35 | 1.12 | 1.255 |
| 15 | CLBP | 31 | F | 30.48 | low-moderate | 7 | 7.85 | 8.05 | 1.28 | 1.11 |
| 16 | CLBP | 31 | F | 22.66 | heavy | 7 | 7.975 | 7.4 | 1.145 | 1.285 |
| 17 | CLBP | 35 | F | 25.23 | heavy | 6 | 6.415 | 12.35 | 0.9 | 1.48 |
| 18 | CLBP | 36 | F | 15.12 | heavy | 7 | 6.3 | 10 | 0.835 | 1 |
| 19 | CLBP | 29 | F | 21.72 | low-moderate | 5 | 7.55 | 6.6 | 1.295 | 1.295 |
| 20 | CLBP | 33 | M | 29.00 | low-moderate | 5 | 8.9 | 7.95 | 1.15 | 0.98 |
| 1 | Healthy | 24 | M | 33.90 | heavy | 0 | 5.72 | 6.3 | 0.98 | 0.98 |
| 2 | Healthy | 31 | M | 25.80 | low-moderate | 0 | 5.45 | 6.9 | 0.81 | 1.155 |
| 3 | Healthy | 28 | M | 21.75 | low-moderate | 0 | 7.85 | 7 | 1.255 | 0.925 |
| 4 | Healthy | 27 | M | 18.62 | low-moderate | 0 | 6.35 | 6.4 | 1.39 | 1.07 |
| 5 | Healthy | 29 | M | 22.07 | low-moderate | 0 | 6.95 | 8.4 | 1.015 | 1.12 |
| 6 | Healthy | 29 | M | 18.83 | low-moderate | 0 | 7.47 | 8.35 | 1.06 | 1.48 |
| 7 | Healthy | 27 | M | 28.41 | Sedentary | 0 | 6.25 | 8.35 | 0.785 | 1.285 |
| 8 | Healthy | 29 | M | 24.53 | low-moderate | 0 | 7.635 | 13.3 | 1.205 | 1.095 |
| 9 | Healthy | 24 | M | 20.52 | low-moderate | 0 | 8.475 | 6.9 | 1.17 | 1.425 |
| 10 | Healthy | 27 | M | 25.71 | low-moderate | 0 | 7.65 | 10.25 | 1.195 | 1.36 |
| 11 | Healthy | 32 | F | 36.88 | low-moderate | 0 | 6.1 | 8.2 | 1.085 | 0.8 |
| 12 | Healthy | 22 | F | 28.55 | low-moderate | 0 | 5.95 | 8.2 | 0.705 | 1.315 |
| 13 | Healthy | 18 | F | 19.88 | Sedentary | 0 | 7.565 | 12.4 | 1.045 | 1.225 |
| 14 | Healthy | 29 | F | 28.16 | heavy | 0 | 6.15 | 4.35 | 1.12 | 1.255 |
| 15 | Healthy | 20 | F | 28.25 | low-moderate | 0 | 7.85 | 8.05 | 1.28 | 1.11 |
| 16 | Healthy | 35 | F | 25.15 | low-moderate | 0 | 7.975 | 7.4 | 1.145 | 1.285 |
| 17 | Healthy | 20 | F | 26.24 | low-moderate | 0 | 6.415 | 12.35 | 0.9 | 1.48 |
| 18 | Healthy | 32 | F | 19.22 | low-moderate | 0 | 6.3 | 10 | 0.835 | 1 |
| 19 | Healthy | 29 | F | 26.85 | low-moderate | 0 | 7.55 | 6.6 | 1.295 | 1.295 |
| 20 | Healthy | 23 | F | 17.22 | low-moderate | 0 | 8.9 | 7.95 | 1.15 | 0.98 |

**List of abbreviations of variables:**

CLBP – Chronic Low Back Pain, LM – Lumbar Multifidus

Gender: M- Male; F- Female

Occupation: 0- Sedentary, 1- Low to Moderate, 2- Heavy

NRS: Numeric Rating Scale
